# Case report: Severe ulcerative dermatitis leading to sepsis in a cat with sporotrichosis by *Sporothrix brasiliensis*

**DOI:** 10.3389/fvets.2025.1573924

**Published:** 2025-04-28

**Authors:** Agustín Cartes, Pamela Thomson, Carlos González, Amanda Ribeiro dos Santos, Rodrigo Díaz, Javiera Puyol, Javiera López

**Affiliations:** ^1^Hospital Clínico Veterinario, Escuela de Medicina Veterinaria, Facultad de Ciencias de la Vida, Universidad Andrés Bello, Viña del Mar, Chile; ^2^One Health Institute, Faculty of Life Sciences, Universidad Andres Bello, Santiago, Chile; ^3^Escuela de Medicina Veterinaria, Facultad de Ciencias de la Vida, Universidad Andrés Bello, Santiago, Chile; ^4^Oak Ridge Institute for Science and Education (ORISE), Oak Ridge, TN, United States

**Keywords:** *Sporothrix brasiliensis*, systemic sporotrichosis, feline sepsis, zoonotic, mycoses

## Abstract

Sporotrichosis caused by *Sporothrix brasiliensis* is an emerging zoonotic mycosis of great clinical relevance in South America. This case highlights its severe systemic presentation, reported for the first time in a feline patient from Chile. A 1-year-old neutered male cat presented with severe ulcerative dermatitis, developing a dysoxic phenotype of sepsis. Diagnosis was made by cytology, histopathology, fungal culture, and polymerase chain reaction, confirming *S. brasiliensis* as the pathogen. Treatment included itraconazole associated with potassium iodide. Despite aggressive antifungal therapy and intensive care, based on fluid resuscitation, optimization of analgesia, and administration of vasoactive drugs, the cat developed refractory hypotension and persistent hyperlactatemia, which ultimately led to euthanasia. This report highlights the high pathogenic potential of *S. brasiliensis* to cause severe systemic disease, even in hosts negative to retroviral infections, and emphasizes the importance of promoting responsible animal management practices to prevent the spread of this infectious agent.

## 1 Introduction

Sporotrichosis is an implantation mycosis caused by dimorphic fungi of the *Sporothrix* genus, which mainly affect subcutaneous tissues, both in humans and animals (1, 2). Globally, *Sporothrix schenckii* has traditionally been the main etiological agent of this mycosis (3). However, in recent decades, *Sporothrix brasiliensis* has emerged as the most virulent and pathogenic species to cats, in South America (4–6). This species has caused major epidemic outbreaks, and is widely distributed in Brazil, although its geographical spread has extended to other countries such as Argentina, Paraguay, and Chile (7, 8). Transmission occurs mainly through bites or scratches, from domestic cats considered the main reservoir and vector of the disease (9, 10). This transmission route has led to the classification of sporotrichosis as a zoonosis, where families with infected cats living in suboptimal socioeconomic and infrastructure conditions, as well as their veterinary professionals and assistants are groups of risk to acquiring this infection (11). Cats infected with this fungus develop nodular and ulcerated skin lesions, predominantly located on the head, limbs, and tail. In severe cases, the infection can spread through the lymphatic system and affect internal organs, complicating clinical management and worsening the prognosis (9, 12). The diagnosis of sporotrichosis is based on a combination of clinical findings, cytology, histopathology, and fungal culture, while molecular techniques are needed for species identification. Species identification is important due the risk of outbreaks in cats linked to the *S. brasiliensis* in South America (13). In Chile, *S. brasiliensis* was first detected in cat case of Sporotrichosis in the Magallanes region in 2022, marking a turning point in the country’s sporotrichosis epidemiology (14). One year later, a human case of lymphocutaneous sporotrichosis was reported in Valparaíso (15), followed by the first documented case in a dog in Santiago (16). More recently, additional cases have been reported suggesting a rapid northward spread of the pathogen the country (17).

### 1.1 Case description

In June 2024, a cat domestic longhair (DLH), 1-year-old, 3.9 kg, neutered male cat from Santo Domingo, Valparaíso (33°38′00″S, 71°39′00″W) was presented to the emergency department of the Veterinary Clinical Hospital, Universidad Andrés Bello (Viña del Mar, Valparaíso, Chile), with ulcerative skin lesions that had developed over weeks and were progressively worsening due to lack of treatment. The cat had an outdoor lifestyle and had no history of travel outside region.

**Figure 1:**
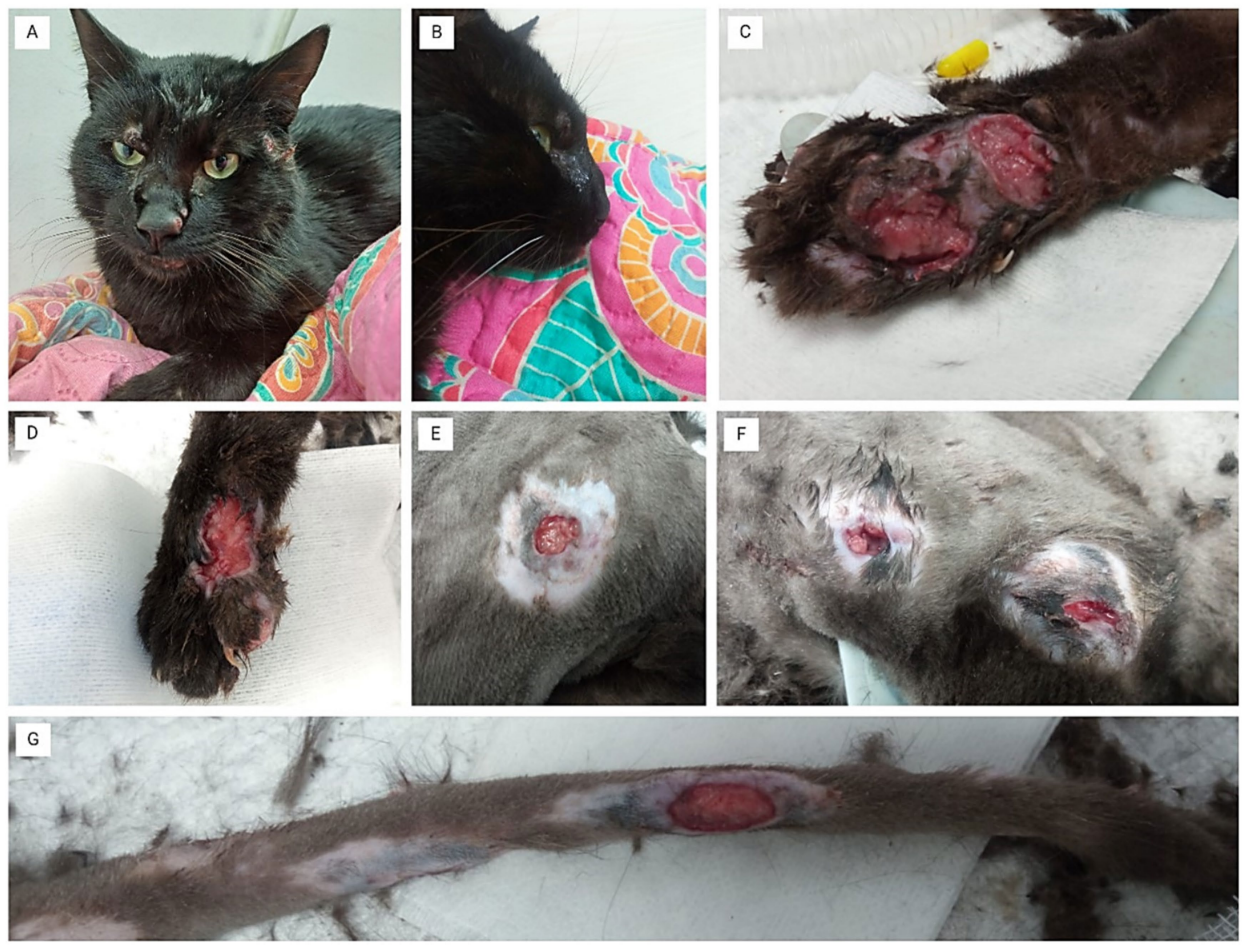
Clinical presentation of ulcerative skin lesions. **(A)** The cat shows facial lesions with deformation of the nasal septum. **(B)** Close-up of the cat’s head, highlighting the nasal plane deformity. **(C)** Severe ulcerative lesions on the left forelimb, with extensive tissue necrosis and deep ulceration. **(D)** Ulcerative lesion on the right forelimb, showing signs of necrosis and suppuration. **(E)** Circular ulcerative lesion on the thorax, with central necrosis and surrounding inflammation. **(F)** Multiple ulcerative lesions on the lateral thoracic area, with signs of necrosis and inflammation around each lesion. **(G)** Ulcerative lesion on the tail, exhibiting severe tissue damage and exposed necrotic tissue.

On the day of admission, June 24, 2024, the patient revealed apathy, moderate dehydration, pale mucous membranes, and multiple areas of subcutaneous ulceration, some of them with necrosis and suppuration (Figures 1C–G). The cat presented pain on palpation of the affected areas, highlighting nasal stridor, along with a visible deformity of the nasal septum and epiphora (Figures 1A,B).

Blood tests showed moderate anemia, thrombocytopenia, hypoglycemia, elevated liver enzymes (ALT and AST), hypokalemia, and hypochloremia (Supplementary Table S1), all indicators of metabolic deterioration linked to systemic inflammation. An abdominal ultrasound revealed bilateral nephropathy, acute inflammatory liver disease, cholecystitis with biliary sludge, and pancreatitis, suggesting a systemic infectious process. Additionally, the patient presented negative results for retroviral infections. FeLV/FIV Sensitivity-Specificity test 94.7%–96.3% and 99.2%–98.9%, respectively, (Speed Duo FeLV / FIV, Virbac, Cerrillos, Chile).

### 1.2 Diagnosis

**Figure 2:**
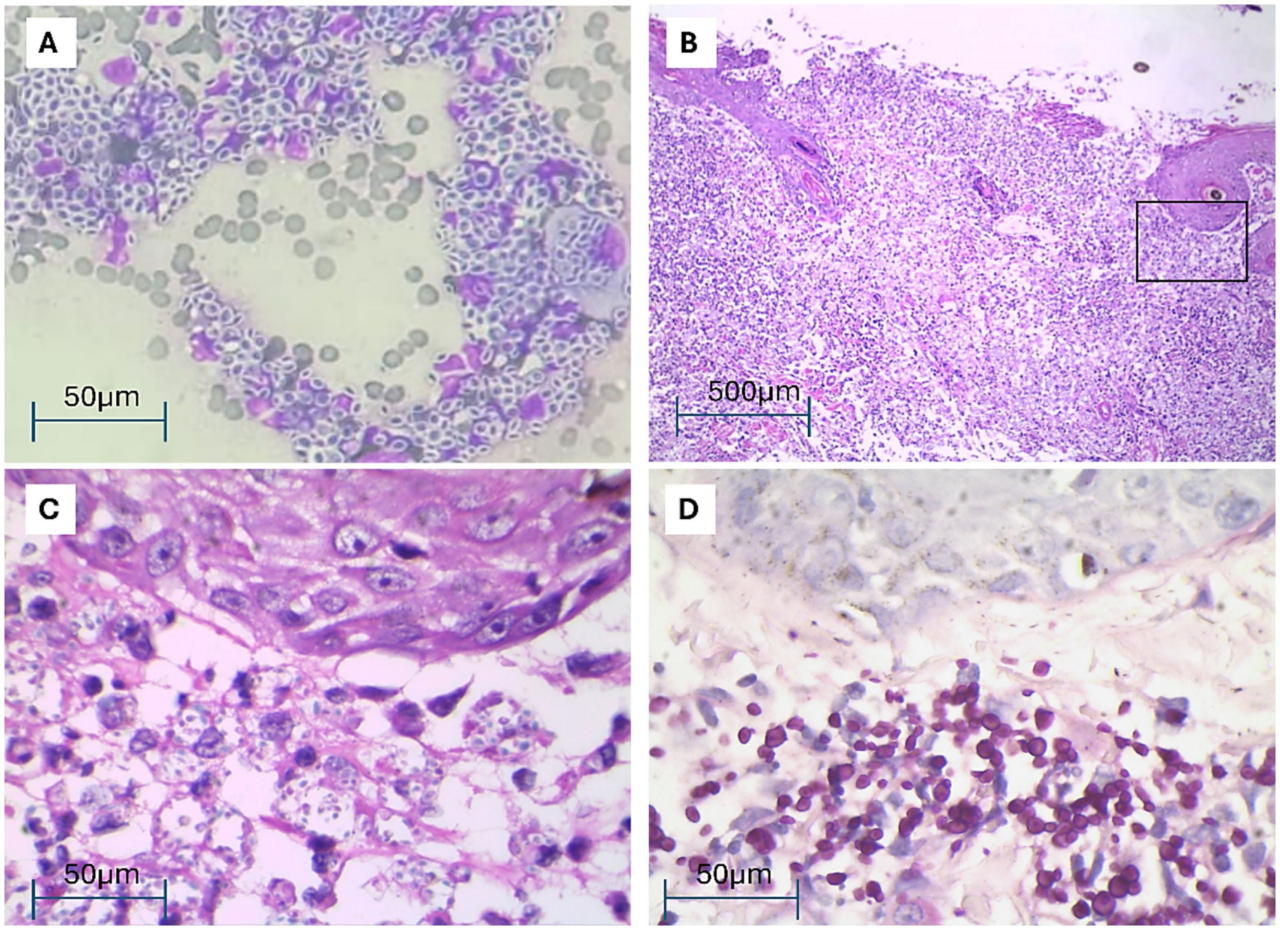
Cytological and histopathological examination of cat skin lesions. **(A)** Cytological smear showing a mixed inflammatory infiltrate, predominantly consisting of macrophages with numerous free or phagocytosed yeast-like organisms, DQ 1000×. **(B)** Skin biopsy showing ulceration, intense and pyogranulomatous inflammatory infiltration affecting dermis and subcutaneous, HE 100×. **(C)** Magnification of an area (black rectangle) of **(B)** showing under the epithelium numerous free or phagocytosed yeast-like organisms, HE 1000. **(D)** Showing under the epithelium numerous PAS positive free or phagocytosed yeast-like organisms, PAS 1000×.

Cytology was performed on the wounds and nasal secretion with samples obtained by imprint and swab, respectively. The samples revealed an abundant amount of yeast (Figure 2A). On the same day it was decided to take tissue biopsy samples for histopathological and microbiological analysis. Histopathological results of the superficial dermis showed an intense diffuse leucocytic infiltration with extensive coalescent inflammatory foci composed of central neutrophils surrounded by lymphocytes and macrophages. The lesions contained numerous cellular debris, representing apoptotic or necrotic components. Numerous PAS-positive yeast-like structures were identified, with morphology suggestive of *Sporothrix* spp. located both intracellularly within the cytoplasm of the macrophages and extracellularly, with irregular sizes and slight pleomorphism. Chronic pyogranulomatous inflammatory process, consistent with fungal infection and few bacteria (Figures 2B–D).

**Figure 3:**
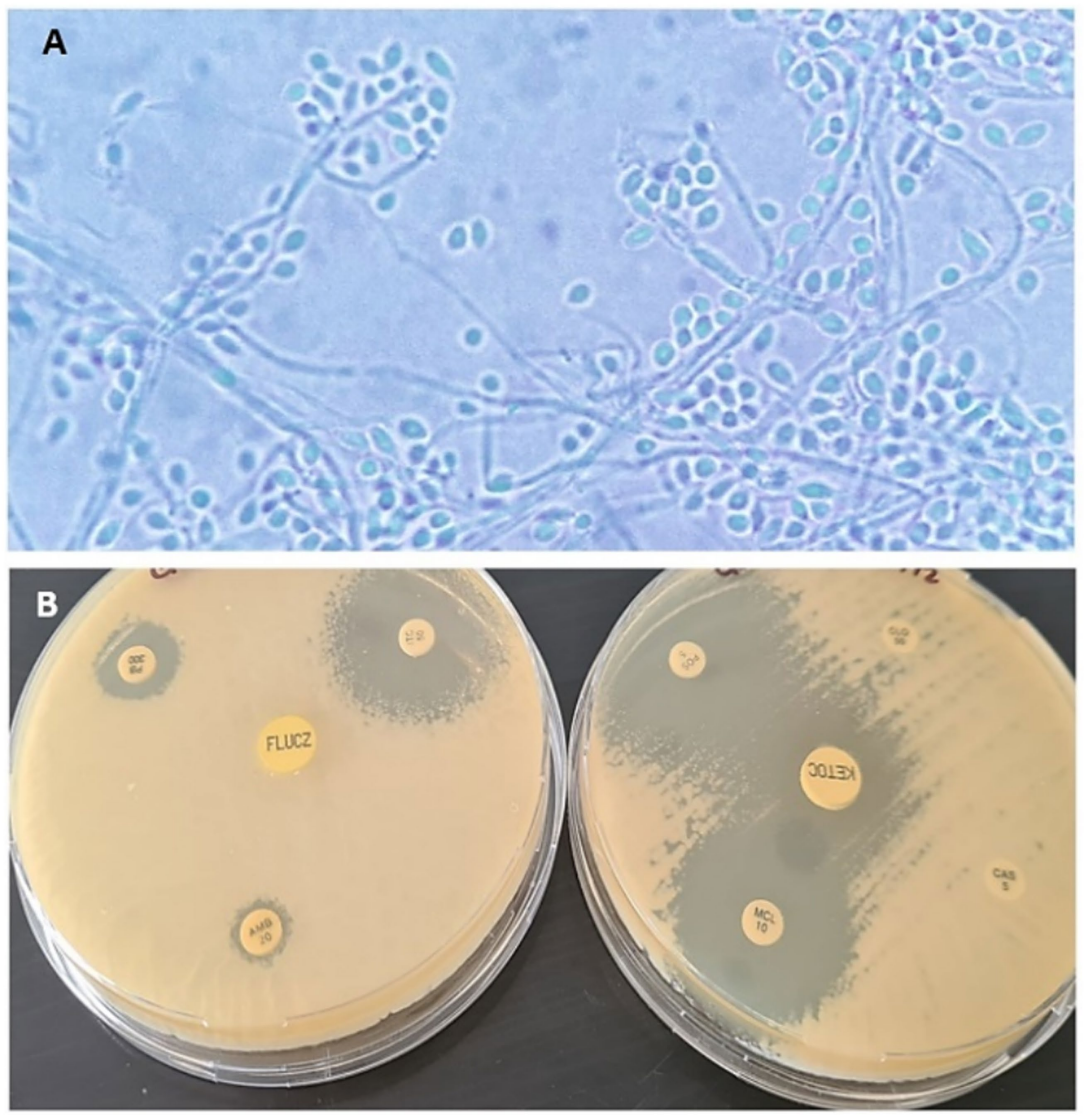
**(A)** Microscopic visualization with 100× magnification: thin mycelia, septate hyaline hyphae, sessile, and sympodial conidia arranged at the end of a thin hypha. **(B)** Susceptibility to antifungals performed by disk diffusion, show halos greater than 18 mm for miconazole, itraconazole, ketoconazole, and posaconazole.

For the mycological study, the tissue sample was then ground and plated on Sabouraud glucose agar (SGA) with chloramphenicol (0.05 g/L) and cycloheximide (0.4 g/L) (Merk, Rahway, NJ, United States), and incubated at 25°C and 37°C for 10 days. Macro and microscopic characteristics allowed the morphological identification of the genus *Sporothrix* (Figure 3A). Additionally, DNA was extracted directly from a subculture of the same colony using the Quick-DNA Fungal/Bacterial kit (Zimo Research, Irvine, CA, United States), according to the manufacturer’s protocol. For the mycological study, the tissue sample was then ground and plated on Sabouraud glucose agar (SGA) with chloramphenicol (0.05 g/L) and cycloheximide (0.4 g/L) (Merk, Rahway, NJ, United States), and incubated at 25°C and 37°C for 10 days. Macro and microscopic characteristics allowed the morphological identification of the genus *Sporothrix* (Figure 3A). Additionally, DNA was extracted directly from a subculture of the same colony using the Quick-DNA Fungal/Bacterial kit (Zimo Research, Irvine, CA, USA), according to the manufacturer’s protocol. The calmodulin and *β*-tubulin were amplified by PCR (18). PCR products were purified and sequenced at the genomics laboratory (Universidad Austral de Chile, Valdivia, Chile) using an ABI3500 sequencer (Applied Biosystems). Sequences were edited using SeqMan® v. 1.0. 7.0.0 (DNAStar Lasergene, Madison, WI, United States) to obtain the consensus sequence, which was compared with the National Center for Biotechnology Information (NCBI) database using the BLASTn tool. A percentage of identity and coverage of 100% for the calmodulin gene allowed the identification of *Sporothrix brasiliensis* (Access code: PQ720582). The most similar calmodulin sequence publicly available in NCBI GenBank was a *S. brasiliensis* strain (Accession OL770362.1) isolated from a feline case in Minas Gerais, Brazil. For the *β*-tubulin gene sequence, the percentage of identity and coverage were 100% and 82%, respectively, and confirmed the identification of *Sporothrix brasiliensis* (Access code: PQ720583). The most similar β-tubulin sequence publicly available in NCBI GenBank was a *S. brasiliensis* strain (Accession OP545816.1) isolated from a patient case Rio de Janeiro, Brazil. The minimum inhibitory concentration (MIC) was determined by broth microdilution, according to document M38-A2 from the Clinical and Laboratory Standards Institute (CLSI standard M38). The drugs tested were amphotericin B (AMB), fluconazole (FLC), itraconazole (ITR), ketoconazole (KET), posaconazole (POS), voriconazole (VRC) and terbinafine (TRB) (Sigma-Aldrich, St. Louis, MO, United States). Briefly, inoculum of each strain of 1–5 × 10^4^ conidia/mL were prepared in sterile saline solution, including strains. MICs were determined by visual inspection after 48–72 h of incubation at 35°C (CLSI standard M38) (19). The azoles demonstrated good activity against the tested strains, showing MIC > 0.25 μg/mL, with exception fluconazole was showed MIC > 128 μg/mL. AMB showed MIC 4 μg/mL, results consistent with those observed with the disk diffusion technique (Figure 3B).

### 1.3 Treatment

The patient was hospitalized on June 24 and transferred to an isolation area where strict management protocols and personal protective equipment were implemented for clinical staff.

The fluid restoration was initiated by calculating dehydration deficits, maintenance requirements, and additional losses, using lactated Ringer’s solution as the crystalloid of choice. Based on the findings of the cytological analysis and the exudative nature of the lesions, associated with respiratory signs and deformation of the nasal septum, empirical therapy was started with itraconazole (100 mg/cat PO SID; Ascend Laboratories, Santiago, Chile) plus potassium iodide (2.5 mg/kg PO; Ahumada, Viña del Mar, Chile). Additionally, ampicillin-sulbactam (20 mg/kg IV TID; Vitalis, Bogotá, Colombia) was incorporated into the therapy. For pain and inflammation management, a combination of tramadol (4 mg/kg IV QID; Vitalis, Bogotá, Colombia), pregabalin (2 mg/kg PO BID; Ascend Laboratories, Santiago, Chile), metamizole (20 mg/kg IV TID; Drag Pharma, Santiago, Chile), and meloxicam (0.1 mg/kg IM SID; baVET, Istanbul, Turkey) were administered. To protect the gastrointestinal mucosa, omeprazole (1 mg/kg IV BID; Vitalis, Bogotá, Colombia) was also included in the treatment regimen, which was continued for 7 days. After 8 days of antifungal therapy, his condition continued to deteriorate, with depression of consciousness, hypothermia, and painful facial abscesses. To optimize analgesia, a continuous ketamine infusion was started, starting with a loading dose of 0.5 mg/kg IV (Troy Laboratories, Sydney, Australia), followed by a 10 μg/kg/min infusion. Tramadol was replaced by methadone at 0.3 mg/kg IV every 6 h (Fresenius Kabi, Bad Homburg, Germany), resulting in effective pain control. A follow-up ultrasound revealed progression of systemic lesions, with worsening nephropathy and liver disease, along with signs of ulcerative gastritis. Laboratory analyses showed significant deterioration compared with initial results: anemia had worsened, leukocyte parameters were increased, and severe metabolic acidosis indicated ongoing systemic decompensation. In addition, ALT and AST levels were increased, reflecting progressive liver inflammation. Thrombocytopenia persisted and hypoproteinemia was detected, suggesting that protein loss is likely related to systemic inflammation (Supplementary Table S1).

### 1.4 Outcome

On day 20, the patient was categorized as septic with a dysoxic phenotype, based on the presence of hypotension (SBP < 100 mmHg) refractory to resuscitation and persistent hyperlactatemia (>4 mmol/L) (20), in addition to the suppurative foci confirmed by cytology and microbiology, which at this time revealed the presence of fungal elements (21). Despite intensive treatment, which included two blood transfusions, continuous infusions of vasoactive drugs such as norepinephrine 0.05–0.15 μg/kg/min IV (Vitalis, Bogotá, Colombia) and dobutamine 5–10 μg/kg/min IV (PiSA, Guadalajara, Mexico) to stabilize blood pressure, and a continuous infusion (CRI) of hydrocortisone 0.2 mg/kg/h IV (Vitalis, Bogotá, Colombia) to control inflammation and suspected relative adrenal insufficiency, the cat remained clinically unstable (Supplementary Figure S1). On day 21, the cat presented signs of irreversible systemic failure, characterized by persistent hemodynamic instability and systemic inflammatory response syndrome (SIRS) with progressive multisystem deterioration. At this point, the patient was euthanized.

## 2 Discussion

We present a case of severe sporotrichosis in a cat caused by *S. brasiliensis* in Santo Domingo-Valparaíso region (33°38′00″S, 71°39′00″W). The presence of this species has been previously documented in cats (17) and a woman (15) in another locality in the same region. In addition, we add the case of a dog in the Metropolitan region (16) and the description of the first outbreak in cats in the Magallanes region (14), located 2,171 km in a straight line from the present study, which suggests a rapid expansion of *S. brasiliensis*. Likewise, other *Sporothix* species have been previously described in Chile, such as *S. schenckii* (22), *S. globosa* (23), and *S. pallida* (24), indicating that this genus has been present in Chile since 2010.

This patient presented rapid systemic spread, with metabolic and hematological alterations, this clinic presentation underlines the high virulence potential of *S. brasiliensis*, which can cause severe systemic disease. Although retroviral tests were negative, it is important to corroborate these results with molecular techniques to rule out false negatives. In this regard, Miranda et al., point out that cats with sporotrichosis coinfected with retroviruses show immunological alterations and more severe clinical presentations compared to retrovirus-negative cats (25). This has also been observed in human patients (12, 26).

On the other hand, Brazilian studies indicate that 69.2% of cases present stable localized lesions, while 30.8% show disseminated cutaneous forms in cats with poor general condition, often associated with a high fungal load and marked pyogranulomatous inflammation in nasal tissues (27). Diagnostic confirmation was achieved by histopathology and fungal culture, the latter being the gold standard for the diagnosis of sporotrichosis. While the identification was performed molecularly, techniques consistent with what has been reported in the literature (10, 14, 17, 28). In this feline patient, itraconazole combined with potassium iodide was administered as a primary antifungal treatment. The use of itraconazole as a first-line antifungal for feline sporotrichosis is justified by its proven efficacy in reducing the fungal load, essential to control the progression of the disease and minimize the risks of transmission; in addition, its safety profile is superior to that of ketoconazole and amphotericin B (29). Some authors recommend potassium iodide as an adjunct to itraconazole in cases unresponsive or resistant to monotherapy, especially in those with respiratory, nasal, or systemic involvement (9, 10, 30). Despite this association, the patient developed a severe systemic syndrome. In this regard, Nakasu et al. (30), highlight the importance of personalized treatment protocols that incorporate antifungal susceptibility testing. Other case reports from Chile support the use of itraconazole as a first-line treatment, but also present its limitations. For example, cases in Santiago with respiratory complications demonstrated mixed results with itraconazole monotherapy; while some cats showed partial recovery, others were euthanized (17). In Magallanes, adjuvant potassium iodide appeared to be beneficial in treating deeper nasal lesions (14).

With the report of this case, we emphasize public education about the risks associated with sporotrichosis and promote responsible animal management practices to prevent the spread of this infectious agent that has come to our country to stay. Probable climate adaptation.

## Data Availability

The datasets presented in this study can be found in online repositories. The names of the repository/repositories and accession number(s) can be found in the article/Supplementary material.
