# Supplementary material for: Case report: Severe ulcerative dermatitis leading to sepsis in a cat with sporotrichosis by Sporothrix brasiliensis
Source: Front Vet Sci. 2025 Apr 28;12:1573924. doi: 10.3389/fvets.2025.1573924 (PMC12067789; doi:10.3389/fvets.2025.1573924)
Supplement: Supplementary file 1 [file Data_Sheet_1.docx]

|  | **At Admission** | **At Euthanasia** | **Mean** |
| --- | --- | --- | --- |
| Erythrocytes (mm3) | 11,73 | 8,17 | 10,1 |
| Hematocrit (%) | 36 | 30 | 33 |
| Hemoglobin (g/dl) | 11 | 8 | 10 |
| Leukocytes (mm3) | 14.110 | 24.480 | 20.009 |
| Segmented Neutrophils (mm3) | 8.466 | 20.808 | 15.849 |
| Lymphocytes (mm3) | 4.939 | 3.427 | 3.296 |
| Eosinophils (mm3) | 282 | 245 | 220 |
| Monocytes (mm3) | 423 | 613 | 613 |
| Platelets (mm3) | 191.000 | 50.000 | 10.2143 |
| Total Protein (g/dl) | 7,80 | 6,50 | 7,37 |
| Albumin (g/dl) | 1,84 | 1,47 | 1,71 |
| Globulin (g/dl) | 5,96 | 5,03 | 5,66 |
| Cholesterol (mg/dl) | 138,00 | 115,00 | 143,14 |
| Phosphorus (mg/dl) | 6,86 | 5,33 | 5,39 |
| Glucose (mg/dl) | 91,60 | 105,80 | 121,61 |
| Creatinine (mg/dl) | 0,63 | 0,29 | 0,47 |
| NUS (mg/dl) | 13,70 | 15,88 | 12,95 |
| ALT (U/L) | 23,10 | 43,40 | 25,67 |
| AST (U/L) | 32,10 | 71,70 | 37,41 |
| AP (U/L) | 15,00 | 5,00 | 14,00 |
| GGT (U/L) | 5,80 | 5,80 | 5,80 |
| Total Bilirubin (mg/dl) | 0,32 | 0,11 | 0,10 |
| Calcium (mg/dl) | 8,06 | 9,84 | 10,27 |
| pH | 7,36 | 7,32 | 7,40 |
| pCO2 (mmHg) | 65,10 | 81,30 | 59,05 |
| pO2 (mmHg) | 63,00 | 40,00 | 61,33 |
| Na+ (mmol/L) | 155,80 | 140,60 | 149,92 |
| K+ (mmol/L) | 4,35 | 2,51 | 4,16 |
| Cl- (mmol/L) | 110,50 | 97,00 | 109,43 |
| Ca++ (mmol/L) | 1,51 | 1,18 | 1,37 |
| Glucose (mmol/L) | 8,82 | 1,80 | 5,79 |
| Lactate (mmol/L) | 3,16 | 1,12 | 2,17 |
| cHCO3- (mmol/L) | 36,60 | 41,60 | 35,70 |
| BE(b) (mmol/L) | -1,30 | -2,80 | -0,05 |

**Table supplementary 1**. Laboratory data during hospitalization. Hematological, biochemical, and blood gas parameters are presented for the patient at two critical time points: upon admission and at the time of euthanasia. The mean values calculated between these two points are also included. The table highlights the progressive deterioration of the patient's clinical condition.

**
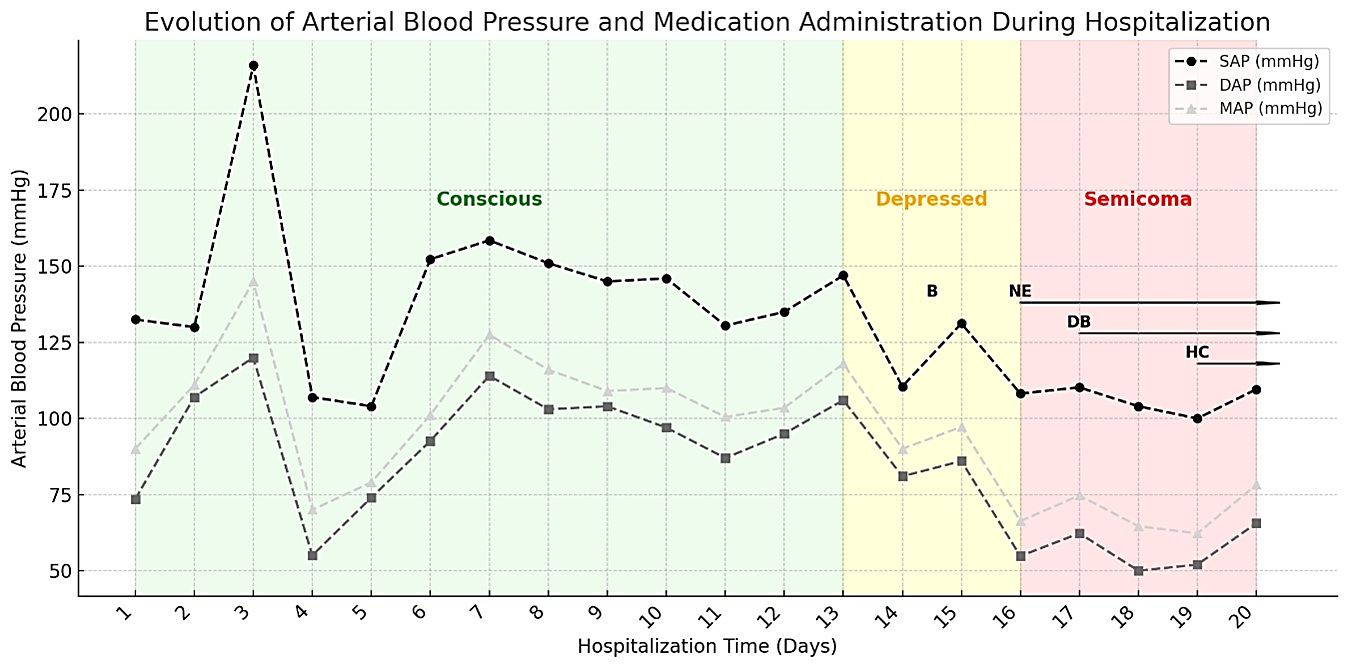
**

**Figure Supplementary 1**. Arterial blood pressure dynamics. The patient's systolic, diastolic, and mean arterial pressures (SAP, DAP, MAP) are plotted over the course of hospitalization, with distinct phases marked by changes in consciousness levels. The administration of the bolus of 10 mL/Kg of crystalloids (B), norepinephrine (NE), dobutamine (DB), and hydrocortisone (HC) is indicated by arrows, reflecting their respective durations.
